# Therapeutic options for the treatment of post-acute sequelae of COVID-19: a scoping review

**DOI:** 10.1186/s12879-025-11131-x

**Published:** 2025-05-22

**Authors:** Yu Bin Seo, Yu Jung Choi, Jun-Won Seo, Eun Jung Kim, Jacob Lee, Joon Young Song

**Affiliations:** 1 Division of Infectious Disease, Department of Internal Medicine, Kangnam Sacred Heart Hospital, Hallym University College of Medicine, Seoul, Republic of Korea; 2 Division of Infectious Diseases, Department of Internal Medicine, Korea University College of Medicine, Seoul, Republic of Korea; 3 Vaccine Innovation Center-KU Medicine, Seoul, Republic of Korea; 4 Departments of Internal Medicine, Chosun University College of Medicine, Gwangju, Republic of Korea; 5 Health, Welfare, Family and Gender Equality Team, National Assembly Research Service, Seoul, Republic of Korea; 6 Division of Infectious Diseases, Department of Internal Medicine, Korea University Guro Hospital, Korea University College of Medicine, Gurodong-ro 148, Guro-gu, Seoul, 08308 Republic of Korea

**Keywords:** PASC, Long COVID, Post-acute COVID-19 syndrome, Therapeutic agents

## Abstract

**Objectives:**

This scoping review aimed to summarize the available studies to address the question of which therapeutic agents can be utilized for patients with post-acute sequelae of COVID-19 (PASC).

**Methods:**

We conducted a systematic search in medical databases, including PubMed and Embase, for studies aligned with our objectives published between January 1, 2020, and July 22, 2024. For each study, we summarized the main symptoms targeted, study design, therapeutic regimens, evaluation tools, and clinical outcomes.

**Results:**

A total of 413 studies were identified, and 39 studies were included in this review based on relevance to the research objectives. We primarily focused on high-level evidence studies, such as meta-analyses and randomized controlled trials, but observational studies were included when evidence was scarce. Therapeutic agents evaluated included hyperbaric oxygen, ivermectin, metformin, naltrexone, micronutrient supplements, antifibrotic agents, antiviral agents, and selective serotonin reuptake inhibitors (SSRIs). Among these, hyperbaric oxygen, antifibrotic agents, antiviral agents, and SSRIs demonstrated promising results. However, the heterogeneity of PASC symptoms posed challenges in synthesizing findings for specific symptom-based outcomes.

**Conclusion:**

Given the heterogeneity of symptoms, this review highlights the need for standardized and targeted research to better address the diverse therapeutic needs of patients with PASC.

**Clinical Trial:**

Not applicable.

**Supplementary Information:**

The online version contains supplementary material available at 10.1186/s12879-025-11131-x.

## Introduction

Although the World Health Organization (WHO) has declared the end of the Coronavirus disease 2019 (COVID-19) pandemic, it remains an important concern that patients continue to experience symptoms after SARS-CoV-2 infection. The mechanisms of these symptoms, referred to various term such as “Long COVID”, “Long post-COVID symptoms”, “Post-acute sequelae of COVID-19 (PASC)” and “Post-COVID-19 syndrome”, have not yet been clearly defined. WHO defined PASC as the continuation or development of new symptoms 3 months after the initial SARS-CoV-2 infection, with these symptoms lasting for at least 2 months with no other explanation [1]. Other organizations, such as the Centers for Disease Control and Prevention (CDC) and the National Institutes of Health, have defined it as symptoms lasting for more than 4 weeks [2, 3]. PASC includes a wide range of symptoms, such as fatigue, cough, shortness of breath, cognitive dysfunction, and myalgic encephalomyelitis/chronic fatigue syndrome (ME/CFS).

The CDC reported that, as of March 2024, approximately 6.7% of adults in the U.S. were experiencing PASC [4]. In South Korea, among confirmed COVID-19 patients, 39.9% visited healthcare facilities due to newly developed condition within 3 months post-infection [5]. Despite the high prevalence of PASC, management of the PASC remains difficult and complex. Recently, there have been some guidelines that recommend various medications and rehabilitation for certain symptoms [6–8]. Nevertheless, it is still challenging due to lack of robust clinical data and the ambiguity in the diagnosis of PASC.

Therefore, we aimed to provide a narrative scoping review of specific medications for PASC that includes the latest insights. This review summarizes the latest evidence on potential medications and offers treatment options for clinicians to use in their actual practice.

## Methods

To achieve clarity and transparency and to avoid poor reporting, we used the Preferred Reporting Items for Systematic Reviews and Meta-Analyses extension for Scoping Reviews (PRISMA-ScR) [9]. Given the rapidly evolving nature of PASC research, we aimed to capture a wide range of emerging evidence, including exploratory studies that might have been excluded under a pre-planned protocol. Our approach adhered to PRISMA-ScR guidelines to ensure methodological transparency and comprehensiveness (Supplementary Table 1). Additionally, we applied the Joanna Briggs Institute (JBI) Critical Appraisal Checklist to qualitatively assess the methodological rigor of included studies, evaluating aspects such as sample size, blinding, and control measures where applicable. The quality assessment of the literature was conducted independently by two researchers. Any agreements and disagreements between the evaluators were then reconciled and analyzed through a researcher meeting in which both researchers participated.

### Searching strategy and inclusion/exclusion criteria

We searched scientific and medical databases (PubMed, Embase, and Cochrane library) for relevant studies published between January 1, 2020, and July 22, 2024. The database search was conducted on the July 22, 2024. We restricted the search language to English only. The following keywords were used in the databases: long Covid-19 (long COVID, post-acute COVID-19 Syndrome and after COVID) and treatment medications, which are listed in Table 1. We conducted analyses including both meta-analyses and randomized clinical trials, and included observational studies when data were insufficient.

The inclusion criteria were as follows: (1) the articles reported the clinical results including the main symptoms targeted, study design, regimen, evaluation tools, clinical outcomes, total number of participants, specific number of PASC and related death/hospitalization; (2) MeSH (Medical Subject Headings) terms and keywords for methods; (3) English literature. The exclusion criteria were as follows: (1) case reports; (2) no relevant data; (3) gray literature (conference proceedings, dissertations, and theses) (4) unavailable full text. Due to the time-sensitive nature of this research, it was not feasible to access the full texts of all identified papers, and thus, only the most relevant and accessible studies were included in the review. Studies were excluded if, (1) they focused on the treatment effect in the acute phase of COVID-19 rather than long COVID, (2) the full text was not available, or (3) they were laboratory studies rather than clinical studies. Many chemical experiments were initially included because the active ingredients of the treatments were searched together; however, all of these were excluded.

**Table 1:** Keywords of medications

Drugs	Keywords
Hyperbaric oxygen	Hyperbaric Hyperbarics Cell respiration Oxygenation (oxygen, oxygenate, oxygene)
Ivermectin	Ivermectin Ivermectine Ivermectins
Metformin	Metformin Metformine Metformins
Naltrexone	Naltrexone Naltrexone
Omega-3 fatty acids	Fatty acids Omega-3 Omega-3 fatty acids
Micronutrient supplement	Vitamin s Vitamine Vitamins
Antifibrotic Agent	Nintedanib
	Pirfenidone
Antiviral agent	Nirmatrelvir-ritonavir
SSRI	Selective serotonin reuptake inhibitors

### Data extraction and outcomes

Data extraction was conducted by the reviewers (EJK and YBS) in consultation with a senior author (JYS). Descriptive data extracted in this scoping review included the author, main symptoms targeted, study design, regimen, evaluation tools, clinical outcomes.

The goal of study was to synthesize the research results on various treatments mentioned in the PASC and to gain insight into medicine that can be used in actual clinical settings. A scoping review differs from a systematic review in that it does not require an assessment of the risk of bias of the included literature and uses a qualitative analysis that analytically reinterprets the literature rather than synthesizing the results of individual studies into an integrated estimate.

## Results

**Fig. 1:**
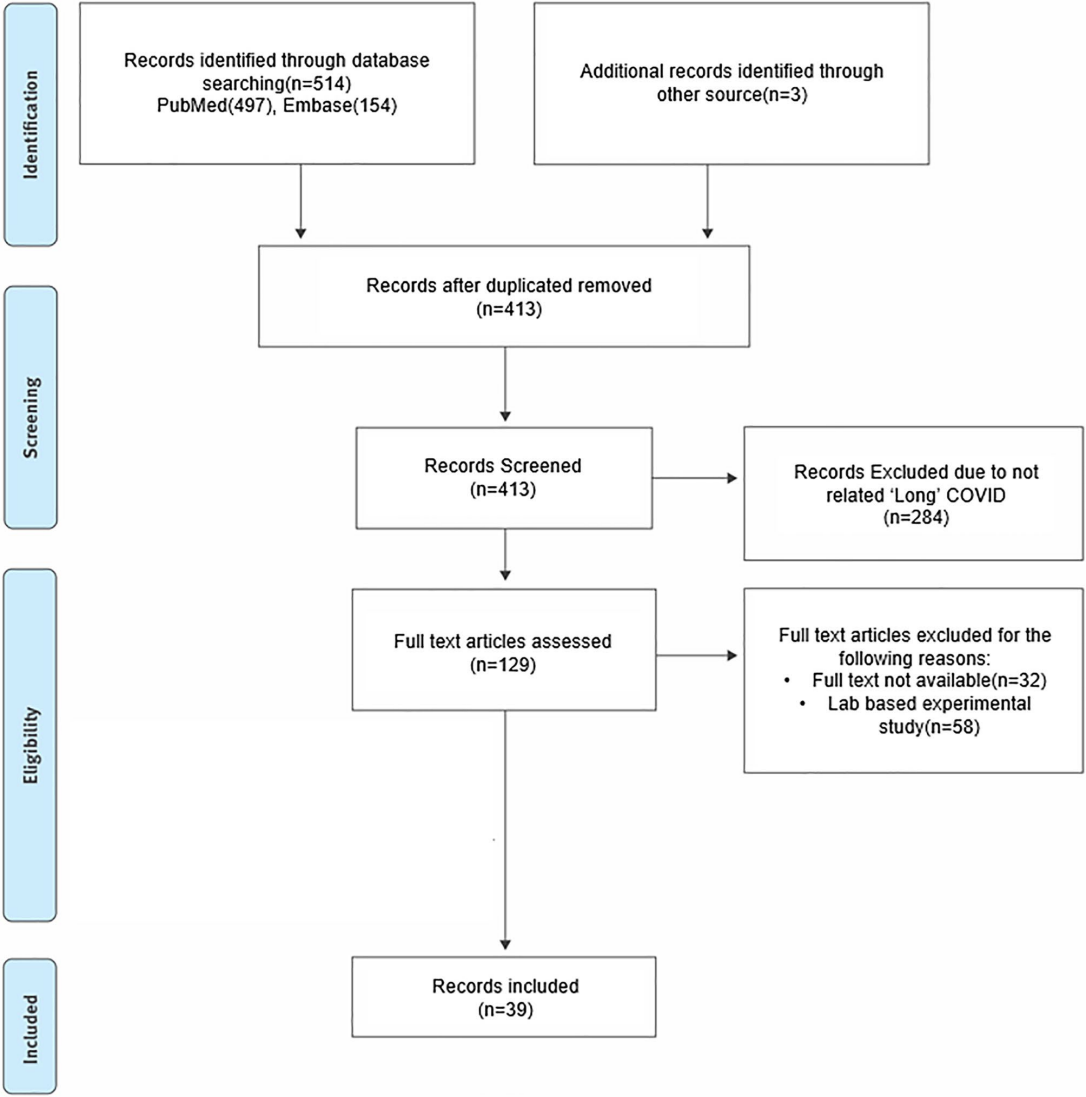
Flowchart of selection process for the scoping review

We identified 517 records, 104 of which were excluded as duplicates. Title and abstract screening were conducted for the remaining 413 articles, 284 of which were excluded because of being unrelated to PASC. For 32 articles, we failed to access the full text due to access limit. Additionally, 58 articles were excluded because they were not clinical trials. Finally, 39 articles were included in the review (Fig. 1). The target symptoms, study design, drug regimen, and outcomes for the therapies included in this scoping review are summarized in Supplementary Tables 2 and 3.

### Hyperbaric oxygen therapy

The mechanism by which hyperbaric oxygen therapy (HBOT) may exert beneficial effects on PASC remains unclear. Generally, HBOT is known to reduce acute phase inflammatory proteins, cytokines, and interleukins, thereby mitigating inflammatory responses. Additionally, it has been reported to enhance growth factors and pro-angiogenic cytokines, thereby promoting the recovery of damaged tissue [10]. Based on these mechanisms, experimental and clinical studies suggest that HBOT may benefit the cognitive system, especially in cases of brain injury or neurodegenerative disease [11]. Recent findings have also reported increases in mitochondrial respiration and mitochondrial mass with HBOT, prompting experimental research into mitochondrial imbalance or dysfunction as a therapeutic target for HBOT [12]. These mechanisms hold promise for alleviating PASC symptoms, particularly those related to brain function and chronic fatigue.

The primary symptoms expected to improve with HBOT in PASC cases are related to cognitive dysfunction, although studies have also explored its effects on fatigue and cardiac function. One study found improvements in cognitive function, attention, executive function, energy level, sleep quality, psychiatric symptoms and pain [13]. Another study reported improvements in quality of life, sleep quality, and reductions in psychiatric and pain symptoms within one month of treatment, with sustained effects observed after one year [14]. Regarding brain function, structural and functional magnetic resonance imaging has shown that HBOT can improve disruptions in white matter tracts and enhance connectivity organization within neural pathways [15]. Cardiac function was also positively affected, with improvements in global work efficiency and normalization of global longitudinal strain, suggesting that HBOT may support the recovery of left ventricular systolic function [16]. However, as all these studies were conducted with fewer than 40 participants, larger-scale studies are necessary to establish more robust evidence for these effects.

In terms of safety, 12 out of 20 patients in one study experienced adverse events (AEs), with a total of 31 AEs reported. Of these, 20 events appeared to be related to HBOT, commonly including cough, chest pain, and chest discomfort; however, no severe AEs were reported, and most were transient [17]. HBOT carries a risk of two main complications: barotrauma to the middle ears or sinuses and pulmonary overpressurization due to pressure effects [18]. Additionally, oxygen toxicity can result in pulmonary and neurological damage, as described by the VENTID acronym (vision changes, ears ringing, nausea, muscle twitching, irritability, and dizziness), as well as ophthalmological complications. Given the current limited evidence, HBOT should be considered with a careful risk-benefit assessment.

### Ivermectin

Ivermectin, used as an antiparasitic agent, has been shown to inhibit viral replication of the SARS-CoV-2 virus in vitro [19]. Although the mechanism of ivermectin is unclear, it is hypothesized that it inhibits viral replication by suppressing the importin α/β1-mediated transport of viral proteins across the nuclear membrane [20]. Based on this mechanism, in the early days of COVID-19 pandemic, ivermectin was considered a promising therapeutic option for COVID-19 treatment. However, in a large randomized controlled study, ivermectin did not reduce hospitalizations or emergency room visits due to SARS-CoV-2 infection [21]. Subsequent studies have not shown ivermectin to be effective against SARS-CoV-2 infection. The efficacy of ivermectin against SARS-CoV-2 infection has not been proven in subsequent studies.

There were few studies evaluating the long-term effects of ivermectin. In the COVID-OUT study, unlike metformin, ivermectin had no effect on the incidence of PASC [22]. In another study, there was no meaningful improvement in recovery, hospital admissions, or long-term results in patients with PASC following ivermectin treatment [23].

Ivermectin has generally been safe to use, with no major side effects reported. However, there is limited research on the relationship between ivermectin and PASC. No studies have demonstrated a clear benefit, making the use of ivermectin controversial. Therefore, administering ivermectin for PASC patients is not reasonable.

### Metformin

Metformin, a biguanide antihyperglycemic drug, is widely used as an oral medication for patients with diabetes. Beyond its blood glucose-lowering effects, it has been found to possess anti-inflammatory properties, raising expectations for its potential usefulness in conditions where excessive inflammatory responses are problematic. In the context of acute respiratory distress syndrome (ARDS) caused by COVID-19, metformin has been reported to inhibit NLRP3 inflammasome activation and IL-1β production in macrophages [24]. Animal studies have further demonstrated its ability to reduce cytokine storms, prevent microvascular damage, and inhibit secondary fibrosis [25]. Based on these findings, clinical studies have reported a reduction in severe infections when metformin is used during the acute phase of COVID-19, leading to various repurposing studies aimed at acute phase treatment [26]. However, clinical studies focusing on the effects of metformin solely in PASC remain scarce.

A study conducted in 2023 investigated the preventive effects of metformin on PASC by organizing patient groups as follows: metformin with ivermectin, metformin with fluvoxamine, metformin with placebo, ivermectin with placebo, fluvoxamine with placebo, or placebo with placebo [22]. Once acute infection was confirmed, metformin was administered at 500 mg on day 1, 500 mg twice daily on days 2–5, and then 500 mg in the morning and 1000 mg in the evening until day 14. Among the 1126 patients observed for nine months, 93 (9.3%) reported PASC, with a hazard ratio for metformin of 0.59 (95% CI 0.39–0.89; *p* = 0.012). No clinically concerning AEs were observed.

Further clinical research is needed to confirm whether metformin effectively prevents PASC. Since this study involved the initiation of metformin treatment during the acute phase of COVID-19, its efficacy specifically for PASC patients remains uncertain. However, as studies have reported no concerning AEs when metformin was administered to adult patients without diabetes, it is plausible that clinical trials focusing exclusively on PASC patients could be conducted, and forthcoming research results are anticipated with interest.

### Naltrexone

Naltrexone, a cyclopropyl derivative is a potent opioid antagonist. Low dose naltrexone (1.5–4.5 mg daily) is known to have immunomodulatory and anti-inflammatory properties, and has been used off-label to reduce symptom severity in conditions such as fibromyalgia, Crohn’s disease, multiple sclerosis, and complex regional pain syndrome [27]. In this context, low-dose naltrexone has been tried to manage PASC. Low-dose naltrexone is hypothesized to alleviate PASC symptoms through neuroinflammation modulation by antagonizing toll-like receptors 4 on microglia and macrophages, leading to reduced production of pro-inflammatory cytokines (e.g., IL-6, TNF-alpha) and oxidative stress [28, 29]. This beneficial impact on neuroinflammation might be linked to the reduction of fatigue, pain, and cognitive impairment in patients with PASC. In addition, low-dose naltrexone may temporarily block opioid receptors, triggering a compensatory increase in endogenous opioid production, which would improve pain sense and mood [30].

According to the observational studies, 52–67% of patients receiving low dose naltrexone reported improvements in PASC symptoms including fatigue, pain, and cognitive function (brain fog, memory and concentration) [29, 31, 32]. In a retrospective cohort study by Tamariz et al., low dose naltrexone showed a hazard ratio of 5.04 for improvement compared with physical therapy alone among 108 patients with PASC (*p* = 0.02) [29]. Low dose naltrexone was well-tolerated with minimal side effects. Only mild fatigue and diarrhea were reported. Across the observational studies, low dose naltrexone consistently showed beneficial therapeutic effects for the PASC patients with substantial improvements in quality of life [29, 31–34].

These studies underscore the potential of low dose naltrexone for managing PASC symptoms, although further randomized controlled trials are essential to validate these findings and establish standardized dosing protocols.

### Palmitoylethanolamide

Palmitoylethanolamide (PEA) is a cannabimimetic compound known for its anti-inflammatory effects and neuroprotective properties. The anti-inflammatory mechanism of PEA primarily involves downregulating mast cells and acting as an agonist for peroxisome proliferator-activated receptor alpha (PPARα), which reduces the production of inflammatory mediators and inhibits the activation of inflammatory cells [35]. Moreover, PEA has been reported to have neuroprotective effects by acting on the mast cell–microglia axis. Considering these mechanisms, PEA becomes a potential therapeutic to improve neruo-inflammation after SARS-coV-2 infection. Recent studies have shown that PEA restores gamma-aminobutyric acid type B (GABAB) receptor activity and cortical plasticity in PASC patients by confirming long-interval intracortical inhibition (LICI) and long-term potentiation (LTP)-like cortical plasticity [36].

Research has provided significant evidence to support the role of PEA in improving olfactory function. Several randomized controlled trials have provided evidence for the efficacy of Palmitoylethanolamide-Luteolin (PEA-LUT) in improving olfactory threshold, discrimination, and identification ability in PASC patients with olfactory dysfunction [37, 38]. Patients who received ultramicronized PEA-LUT (umPEA-LUT) in combination with olfactory training (OT) showed a greater improvement in parosmia symptoms compared to patients who received OT alone [39]. The umPEA-LUT-treated patients showed the greatest improvement in TDI scores (21.8 ± 9.4 to 29.7 ± 7.5, *p* < 0.01) followed by patients on umPEA-LUT with OT (19.6 ± 6.29 to 27.5 ± 2.7, *p* < 0.01). Patients in the combination (umPEA-LUT with OT) and umPEA-LUT groups had significantly improved TDI scores compared to alpha-lipoic acid and control groups (*p* < 0.001). Thus, PEA may be a therapeutic option that can be used in patients with olfactory dysfunction. In addition, umPEA-LUT was shown to significantly improve cognitive dysfunction, supporting the assertion that it may be effective in improving other neuroinflammatory symptoms associated with PASC. Treatment with co-ultraPEA-LUT showed a significant improvement in Prospective–Retrospective Memory Questionnaire (PRMQ) score (T0: 51.94 ± 10.55, T1: 39.67 ± 13.02, *p* < 0.01) and Montreal Cognitive Assessment (MoCA) raw score (T0: 25.76 ± 2.3, T1: 27.2 ± 2, *p* = 0.026) [40].

Although its effect on improving olfactory dysfunction, no significant improvement in olfactory function was observed in patients on PEA-LUT alone without OT [41]. OT remains the cornerstone of treatment for patients with olfactory dysfunction after SARS-CoV-2 infection and PEA could be a potential supplementary treatment with its safety confirmed in these trials. In addition to improving olfactory function, PEA has shown efficacy in alleviating other general PASC symptoms in observational studies [42, 43]. A large-scale randomized controlled trial is warranted to investigate this aspect further.

### Micronutrient supplement

Historically, the effects of vitamins in infectious diseases have been a topic of extensive discussion. While the theoretical basis for their positive outcomes remains partially understood, several positive findings have been reported. Vitamin A enhances antibody responses by increasing lymphocyte numbers [44]. Vitamin B, although its effects vary by subtype, is generally involved in the cytotoxic immune response of natural killer cells and CD8 + T cells [45]. Vitamin C is known for its antioxidant and anti-inflammatory effects, while vitamin D plays a crucial role in the maturation of immune cells through its receptors on B cells, T cells, and antigen-presenting cells, alongside its immunomodulatory effects [46, 47]. Vitamin E, fundamentally recognized for its antioxidant properties, also exhibits anti-inflammatory effects and immunomodulatory functions [48]. Based on these existing foundations, numerous studies have investigated the therapeutic potential of these vitamins in COVID-19 and their role in preventing PASC. Additionally, organic compounds such as alpha-lipoic acid and L-arginine have been included in such studies. However, research on their efficacy when administered in PASC conditions remains limited. Randomized controlled trials focusing on single compounds are rare, with studies often conducted using compound mixtures or observational designs, making it challenging to isolate the pure therapeutic effects of these agents in PASC.

A randomized controlled trial conducted on PASC patients experiencing persistent fatigue evaluated the effects of a mixture of L-arginine and vitamin C (1.66 g L-arginine plus 500 mg liposomal vitamin C) administered for one month. The study demonstrated a significant improvement in the 6-minute walk distance after one month (40.5 m; placebo: 75 m, *p* = 0.001) and handgrip strength (7.5 kg compared to placebo: 6.6 kg, *p* = 0.03). Furthermore, endothelial function, assessed through brachial artery dilation following a transient period of forearm ischemia, showed notable improvement (4.3% vs. 9.4%, *p* = 0.03) [49]. Conversely, in a study involving PASC patients with persistent anosmia for over three months, alpha-lipoic acid (300 mg, twice daily for three months) was used as an adjuvant to olfactory training. The study found no significant differences between the treatment and control groups in the Connecticut Chemosensory Clinical Research Center (CCCRC) score (*p* = 0.63), olfactory threshold (*p* = 0.50), identification score (*p* = 0.96), or Visual Analog Scale score (*p* = 0.97) [50].

A meta-analysis investigating the preventive effects of vitamin supplementation during the acute phase of COVID-19 on the development of PASC concluded that no definitive conclusions could be drawn [51]. The lack of sufficient studies, heterogeneity in research design, and the generally low quality of the available data underscore the need for further investigation. Similarly, studies on the effectiveness of micronutrient supplementation in the PASC phase remain insufficient. Given the relatively low risk of AEs associated with micronutrient supplementation, there is a pressing need for well-designed, objective studies to provide better guidance for clinical practice.

### Antifibrotic agents

Acute respiratory distress syndrome was reported to develop in 42% of patients with COVID-19 pneumonia, and 61–81% of those requiring intensive care [52]. Pulmonary fibrosis has been identified as a significant long-term complication of severe COVID-19, particularly in patients with ARDS. Several studies have reported beneficial effects of antifibrotic agents, particularly pirfenidone and nintedanib, in the treatment of pulmonary fibrosis associated with severe COVID-19 and PASC [53–56]. Nintedanib inhibits pathways involving fibroblast growth factor, vascular endothelial growth factor, and platelet-derived growth factor, thereby reducing fibrosis and inflammation, while pirfenidone targets pro-fibrotic cytokines such as TGF-β and TNF-α, decreasing fibroblast activation and collagen synthesis [55].

In a randomized study comparing the efficacy and safety of two antifibrotic agents, in treating lung fibrosis in patients with PASC (*n* = 30), both nintedanib and pirfenidone significantly improved pulmonary function (forced vital capacity and diffusing capacity), 6-minute walk test (6MWT) distances and oxygen saturation levels over 12 weeks [53]. In particular, nintedanib showed greater improvement in 6MWT distance (*p* = 0.02) and oxygen saturation levels (*p* = 0.005), but accompanied by more frequent adverse events including diarrhea (80%) and nausea/vomiting (66.6%). In a retrospective cohort study of 5,000 patients receiving pirfenidone or nintedanib, pulmonary function parameters were preserved at baseline levels after 12 months [54]. Nintedanib showed higher rates of gastrointestinal adverse events. Another retrospective cohort study analyzed the impact of antifibrotic agents on 1-year mortality among COVID-19 patients with acute respiratory failure [55]. Patients in the antifibrotic group had a significantly higher survival probability compared to the control group (84.42% vs. 69.87%, hazard ratio 0.434 [95% CI 0.264–0.712]). Nintedanib demonstrated a statistically significant reduction in 1-year mortality compared to the control group (*p* = 0.013), while pirfenidone improved survival rates without achieving statistical significance (*p* = 0.601). A single-center, prospective observational study evaluated the efficacy of nintedanib and pirfenidone in managing lung fibrosis when combined with steroids (steroids alone vs. steroid plus pirfenidone vs. steroid plus nintedanib) [56]. Nintedanib demonstrated a significantly greater improvement in the chest computed tomography severity score compared to pirfenidone and steroids alone.

Early administration of antifibrotic agents might be associated with better outcomes in lung function and fibrosis resolution in patients with PASC [53–56]. The studies highlight the importance of antifibrotic therapy as part of a multidisciplinary approach to the management of COVID-19-induced pulmonary fibrosis and emphasize the need for further large-scale randomized controlled trials to refine treatment strategies. Although some studies have reported that these treatments are associated with GI problems, they can still be used with caution.

### Anti-viral agents

The mechanism by which early antiviral treatment prevents PASC remains unclear. It is hypothesized that the SARS-CoV-2 virus infection leads to PASC by sustaining inflammation in the body over an extended period [57, 58]. In this perspective, early antiviral treatment inhibits viral proliferation and reduces the viral load in the body, which is expected to improve the symptoms related to COVID-19.

Recent studies have validated the effectiveness of early antiviral administration in preventing PASC. Two meta-analyses of observational studies showed that early administration of antiviral drugs significantly prevented PASC compared with non-antiviral treatment [59, 60]. Additionally, early administration of antiviral drugs reduced the risk of PASC-related hospitalization and death [60].

Observational studies have shown a reduction in PASC with remdesivir [61, 62], but randomized clinical trials have not shown a significant difference, indicating no clear long-term benefit of remdesivir [63]. Oral antivirals demonstrated more favorable outcomes in observational studies, which confirmed the effects of both nirmatrelvir/ritonavir (NMV-r) and molnupiravir in reducing PASC incidence, hospitalization, and death [64–66]. Individual studies have shown significant beneficial effects of oral antiviral agents on long-term cardiovascular risk [67] and neuropsychiatric sequelae [68]. On the other hand, in the target trial emulation study comparing matched cohorts receiving NMV-r versus no treatment, no significant differences were observed in the incidence of most post-COVID-19 conditions (29 out of 31), except for venous thromboembolism and pulmonary embolism [69].

It is reasonable to assume that early antiviral treatment would be effective in preventing PASC. However, there are limitations, as most of the studies included in the meta-analysis are observational and rely on health insurance data. Additionally, studies on the effectiveness of antiviral agents after a PASC diagnosis are insufficient, and research on their safety profile remains limited. A recently published double-blind randomized clinical trial, the STOP-PASC study, which evaluated the efficacy of a 15-day course of NMV-r in patients with PASC, also failed to demonstrate any significant benefit [70]. Thus, the efficacy of antiviral therapy in patients with PASC is questionable and requires further study.

### Selective serotonin reuptake inhibitors

Selective serotonin reuptake inhibitors (SSRIs) are hypothesized to reduce the risk of PASC through several key mechanisms, including immunomodulation, antiplatelet effect, enhancement of vagal activity, and neurotransmitter regulation [71, 72].

In a retrospective study using data from the National COVID Cohort Collaborative (N3C) including 17,908 patients, SSRIs with sigma-1 receptor (S1R) agonist activity (e.g., fluvoxamine, fluoxetine, escitalopram) had a 29% reduction in relative risk (RR) of PASC compared to unexposed patients (RR = 0.704; 95% CI, 0.58–0.85; *p* < 0.001), while Non-S1R Agonist SSRIs (e.g., sertraline, paroxetine) showed a 21% reduction in RR of PASC compared to unexposed patients (RR = 0.79; 95% CI, 0.67–0.93; *p* = 0.005) [73]. In another retrospective study of the N3C database comprising 302,626 patients, SSRI use during acute COVID-19 was associated with a lower risk of PASC (adjusted risk ratio 0.92 [95% CI: 0.86–0.99]) compared to non-SSRI users [72]. However, a systematic review and meta-analysis of 14 clinical studies found that fluvoxamine did not consistently prevent the development of PASC [74]. Nonetheless, some studies have reported reductions in neuropsychiatric symptoms, including fatigue and depression, in patients treated with fluvoxamine. These discrepancies may arise from variations in dosing regimens, timing of administration, and patient characteristics.

Given their anti-inflammatory and neuropsychiatric properties, SSRIs present a promising therapeutic option for PASC. However, to determine their true clinical efficacy, future large-scale, well-controlled randomized trials and mechanistic studies are crucial.

## Conclusion

We conducted a scoping review of nine potential therapeutic options for PASC that are currently under consideration. This is summarized in Supplementary Tables 2 and 3.

In addressing the ongoing challenges posed by PASC, this review provides a comprehensive review of emerging treatment options. Despite significant progress in identifying potential therapeutic strategies, the current landscape highlights critical gaps in robust clinical evidence. There are several limitations to this study. First, this review does not include studies published after the completion of the literature search, suggesting that ongoing updates will be necessary. Additionally, due to time constraints and the need for conciseness, we have focused on eight key medications that have been extensively studied and practical. The treatments mentioned above show promising results in mitigating PASC symptoms, yet they often lack large-scale randomized controlled trials to conclusively validate efficacy and safety. Thus, a deeper understanding of the pathophysiological mechanisms underlying PASC is essential, and future research should prioritize rigorous, multidisciplinary investigations to address these limitations and refine treatment protocols.

## Electronic supplementary material

Below is the link to the electronic supplementary material.

Supplementary Material 1

Supplementary Material 2

Supplementary Material 3

## Data Availability

All data supporting the findings of this study are available within the paper and its Supplementary Information.

## References

[1] World Health Organization. A clinical case definition of post COVID-19 condition by a Delphi consensus, 6 October 2021. World Health Organization: 2021.

[2] Centers for Disease Control and Prevention. Clinical overview of long COVID. 2024.

[3] National Institute for Health and Care Excellence. COVID-19 rapid guideline: managing COVID-19. 2024. 34181371

[4] Centers for Disease Control and Prevention, Long COVID. Household Pulse Survey. National Center for Health Statistics 2024.

[5] Jang TI, Kim JY, Lim HS, Jeon SY, Choi H, Son KJ. Research report of National health insurance service Ilsan hospital: risk of clinical sequelae after the acute phase of SARS-CoV-2 infection. 2022.

[6] Seo JW, Kim SE, Kim Y, Kim EJ, Kim T, Kim T, et al. Updated clinical practice guidelines for the diagnosis and management of long COVID. Infect Chemother. 2024;56:122. 38527781 10.3947/ic.2024.0024 PMC10990882

[7] Venkatesan P. NICE guideline on long COVID. Lancet Respiratory Med. 2021;9:129. 10.1016/S2213-2600(21)00031-X PMC7832375 33453162

[8] Yelin D, Moschopoulos CD, Margalit I, Gkrania-Klotsas E, Landi F, Stahl JP, et al. ESCMID rapid guidelines for assessment and management of long COVID. Clin Microbiol Infect. 2022;28:955–72. 35182760 10.1016/j.cmi.2022.02.018 PMC8849856

[9] Tricco AC, Lillie E, Zarin W, O’Brien KK, Colquhoun H, Levac D, et al. PRISMA extension for scoping reviews (PRISMA-ScR): checklist and explanation. Ann Intern Med. 2018;169:467–73. 30178033 10.7326/M18-0850

[10] De Wolde SD, Hulskes RH, Weenink RP, Hollmann MW, Van Hulst RA. The effects of hyperbaric oxygenation on oxidative stress, inflammation and angiogenesis. Biomolecules. 2021;11:1210. 34439876 10.3390/biom11081210 PMC8394403

[11] Gottfried I, Schottlender N, Ashery U. Hyperbaric oxygen Treatment-From mechanisms to cognitive improvement. Biomolecules. 2021;11:1520. 34680155 10.3390/biom11101520 PMC8533945

[12] Schottlender N, Gottfried I, Ashery U. Hyperbaric oxygen treatment: effects on mitochondrial function and oxidative stress. Biomolecules. 2021;11:1827. 34944468 10.3390/biom11121827 PMC8699286

[13] Zilberman-Itskovich S, Catalogna M, Sasson E, Elman-Shina K, Hadanny A, et al. Hyperbaric oxygen therapy improves neurocognitive functions and symptoms of post-COVID condition: randomized controlled trial. Sci Rep. 2022;12:11252. 35821512 10.1038/s41598-022-15565-0 PMC9276805

[14] Hadanny A, Zilberman-Itskovich S, Catalogna M, Elman-Shina K, Lang E, Finci S, et al. Long term outcomes of hyperbaric oxygen therapy in post Covid condition: longitudinal follow-up of a randomized controlled trial. Sci Rep. 2024;14:3604. 38360929 10.1038/s41598-024-53091-3 PMC10869702

[15] Catalogna M, Sasson E, Hadanny A, Parag Y, Zilberman-Itskovich S, Efrati S. Effects of hyperbaric oxygen therapy on functional and structural connectivity in post-COVID-19 condition patients: A randomized, sham-controlled trial. NeuroImage: Clin. 2022;36:103218. 36208548 10.1016/j.nicl.2022.103218 PMC9528018

[16] Leitman M, Fuchs S, Tyomkin V, Hadanny A, Zilberman-Itskovich S, Efrati S. The effect of hyperbaric oxygen therapy on myocardial function in post-COVID-19 syndrome patients: a randomized controlled trial. Sci Rep. 2023;13:9473. 37301934 10.1038/s41598-023-36570-x PMC10257166

[17] Kjellberg A, Hassler A, Bostrom E, El Gharbi S, Al-Ezerjawi S, Kowalski J, et al. Hyperbaric oxygen therapy for long COVID (HOT-LoCO), an interim safety report from a randomised controlled trial. BMC Infect Dis. 2023;23:33. 36670365 10.1186/s12879-023-08002-8 PMC9854077

[18] Sen S, Sen S. Therapeutic effects of hyperbaric oxygen: integrated review. Med Gas Res. 2021;11:30–3. 33642335 10.4103/2045-9912.310057 PMC8103971

[19] Caly L, Druce JD, Catton MG, Jans DA, Wagstaff KM. The FDA-approved drug Ivermectin inhibits the replication of SARS-CoV-2 in vitro. Antiviral Res. 2020;178:104787. 32251768 10.1016/j.antiviral.2020.104787 PMC7129059

[20] Tessier TM, Dodge MJ, Prusinkiewicz MA, Mymryk JS. Viral appropriation: laying claim to host nuclear transport machinery. Cells. 2019;8:559. 31181773 10.3390/cells8060559 PMC6627039

[21] Reis G, Silva EA, Silva DC, Thabane L, Milagres AC, Ferreira TS, et al. Effect of early treatment with Ivermectin among patients with Covid-19. N Engl J Med. 2022;386:1721–31. 35353979 10.1056/NEJMoa2115869 PMC9006771

[22] Bramante CT, Buse JB, Liebovitz DM, Nicklas JM, Puskarich MA, Cohen K, et al. Outpatient treatment of COVID-19 and incidence of post-COVID-19 condition over 10 months (COVID-OUT): a multicentre, randomised, quadruple-blind, parallel-group, phase 3 trial. Lancet Infect Dis. 2023;23:1119–29. 37302406 10.1016/S1473-3099(23)00299-2 PMC11259948

[23] Hayward G, Yu L-M, Little P, Gbinigie O, Shanyinde M, Harris V, et al. Ivermectin for COVID-19 in adults in the community (PRINCIPLE): an open, randomised, controlled, adaptive platform trial of short-and longer-term outcomes. J Infect. 2024;88:106130. 38431155 10.1016/j.jinf.2024.106130 PMC10981761

[24] Xian H, Liu Y, Rundberg Nilsson A, Gatchalian R, Crother TR, Tourtellotte WG, et al. Metformin Inhibition of mitochondrial ATP and DNA synthesis abrogates NLRP3 inflammasome activation and pulmonary inflammation. Immunity. 2021;54:1463–77. 34115964 10.1016/j.immuni.2021.05.004 PMC8189765

[25] Rosa IF, Pecanha APB, Carvalho TRB, Alexandre LS, Ferreira VG, Doretto LB, et al. Photobiomodulation reduces the cytokine storm syndrome associated with COVID-19 in the zebrafish model. Int J Mol Sci. 2023;24:7104. 37047078 10.3390/ijms24076104 PMC10094635

[26] Reis G, Dos Santos Moreira Silva EA, Medeiros Silva DC, Thabane L, Cruz Milagres A, Ferreira TS, et al. Effect of early treatment with Metformin on risk of emergency care and hospitalization among patients with COVID-19: the TOGETHER randomized platform clinical trial. Lancet Reg Health - Americas. 2022;6:100142. 34927127 10.1016/j.lana.2021.100142 PMC8668402

[27] Toljan K, Vrooman B. Low-Dose Naltrexone (LDN)-Review of therapeutic utilization. Med Sci. 2018;6:82. 10.3390/medsci6040082 PMC6313374 30248938

[28] Lohn M, Wirth KJ. Potential pathophysiological role of the ion channel TRPM3 in myalgic encephalomyelitis/chronic fatigue syndrome (ME/CFS) and the therapeutic effect of low-dose Naltrexone. J Translational Med. 2024;22:630. 10.1186/s12967-024-05412-3 PMC11227206 38970055

[29] Tamariz L, Bast E, Klimas N, Palacio A. Low-dose Naltrexone improves post-COVID-19 condition symptoms. Clin Ther. 2024;46:e101. e106.\. 38267326 10.1016/j.clinthera.2023.12.009

[30] Hatfield E, Phillips K, Swidan S, Ashman L. Use of low-dose Naltrexone in the management of chronic pain conditions: A systematic review. J Am Dent Assoc. 2020;151:891–902. 33228882 10.1016/j.adaj.2020.08.019

[31] Isman A, Nyquist A, Strecker B, Harinath G, Lee V, Zhang X, et al. Low-dose Naltrexone and NAD + for the treatment of patients with persistent fatigue symptoms after COVID-19. Volume 36. Behavior, & Immunity - Health: Brain; 2024. p. 100733. 10.1016/j.bbih.2024.100733 PMC10862402 38352659

[32] Hurt RT, Yadav S, Schroeder DR, Croghan IT, Mueller MR, Grach SL, et al. Longitudinal progression of patients with long COVID treated in a Post-COVID clinic: A Cross-Sectional survey. J Prim Care Community Health. 2024;15:21501319241258671. 38813984 10.1177/21501319241258671 PMC11141226

[33] Bonilla H, Tian L, Marconi VC, Shafer R, McComsey GA, Miglis M, et al. Low-dose Naltrexone use for the management of post-acute sequelae of COVID-19. Int Immunopharmacol. 2023;124:110966. 37804660 10.1016/j.intimp.2023.110966 PMC11028858

[34] O’Kelly B, Vidal L, McHugh T, Woo J, Avramovic G, Lambert JS. Safety and efficacy of low dose naltrexone in a long covid cohort; an interventional pre-post study. Brain, Behavior, & Immunity - Health. 2022;24:100485. 10.1016/j.bbih.2022.100485 PMC9250701 35814187

[35] Noce A, Albanese M, Marrone G, Di Lauro M, Pietroboni Zaitseva A, Palazzetti D, et al. Ultramicronized palmitoylethanolamide (um-PEA): a new possible adjuvant treatment in COVID-19 patients. Pharmaceuticals. 2021;14:336. 33917573 10.3390/ph14040336 PMC8067485

[36] Versace V, Ortelli P, Dezi S, Ferrazzoli D, Alibardi A, Bonini I, et al. Co-ultramicronized palmitoylethanolamide/luteolin normalizes GABAB-ergic activity and cortical plasticity in long COVID-19 syndrome. Clin Neurophysiol. 2023;145:81–8. 36455453 10.1016/j.clinph.2022.10.017 PMC9650483

[37] D’ascanio L, Vitelli F, Cingolani C, Maranzano M, Brenner M, Di Stadio A. Randomized clinical trial olfactory dysfunction after COVID-19: olfactory rehabilitation therapy vs. intervention treatment with palmitoylethanolamide and Luteolin: preliminary results. Eur Rev Med Pharmacol Sci. 2021;25:4156–62. 34156697 10.26355/eurrev_202106_26059

[38] Di Stadio A, D’Ascanio L, Vaira LA, Cantone E, De Luca P, Cingolani C, et al. Ultramicronized palmitoylethanolamide and Luteolin supplement combined with olfactory training to treat post-COVID-19 olfactory impairment: a multi-center double-blinded randomized placebo-controlled clinical trial. Curr Neuropharmacol. 2022;20:2001–12. 35450527 10.2174/1570159X20666220420113513 PMC9886808

[39] Cantone E, D’Ascanio L, De Luca P, Roccamatisi D, La Mantia I, Brenner MJ, et al. Persistent COVID-19 parosmia and olfactory loss post olfactory training: randomized clinical trial comparing central and peripheral-acting therapeutics. Eur Arch Otorhinolaryngol. 2024;1:8. 10.1007/s00405-024-08548-6 PMC11211159 38492007

[40] Cenacchi V, Furlanis G, Menichelli A, Lunardelli A, Pesavento V, Manganotti P. Co-ultraPEALut in subjective cognitive impairment following SARS-CoV-2 infection: an exploratory retrospective study. Brain Sci. 2024;14:293. 38539680 10.3390/brainsci14030293 PMC10968982

[41] De Luca P, Camaioni A, Marra P, Salzano G, Carriere G, Ricciardi L, et al. Effect of ultra-micronized palmitoylethanolamide and Luteolin on olfaction and memory in patients with long COVID: results of a longitudinal study. Cells. 2022;11:2552. 36010630 10.3390/cells11162552 PMC9406356

[42] Pirro M, Ferri L, Piccioni L, Bellucci AM, Bartolucci F, Russo A, et al. What is the role of palmitoylethanolamide Co-Ultramicronized with Luteolin on the symptomatology reported by patients suffering from long COVID? A retrospective analysis performed by a group of general practitioners in a Real-Life setting. Nutrients. 2023;15:3701. 37686733 10.3390/nu15173701 PMC10490268

[43] Raciti L, De Luca R, Raciti G, Arcadi FA, Calabrò RS. The use of palmitoylethanolamide in the treatment of long COVID: a real-life retrospective cohort study. Med Sci. 2022;10:37. 10.3390/medsci10030037 PMC9326613 35893119

[44] Stephensen CB. Vitamin A, infection, and immune function. Annu Rev Nutr. 2001;21:167–92. 11375434 10.1146/annurev.nutr.21.1.167

[45] Partearroyo T, Ubeda N, Montero A, Achon M, Varela-Moreiras G. Vitamin B(12) and folic acid imbalance modifies NK cytotoxicity, lymphocytes B and lymphoprolipheration in aged rats. Nutrients. 2013;5:4836–48. 24288024 10.3390/nu5124836 PMC3875921

[46] Kashiouris MG, L’Heureux M, Cable CA, Fisher BJ, Leichtle SW, Fowler AA. The emerging role of vitamin C as a treatment for Sepsis. Nutrients. 2020;12:292. 31978969 10.3390/nu12020292 PMC7070236

[47] Ismailova A, White JH. Vitamin D, infections and immunity. Reviews Endocr Metabolic Disorders. 2022;23:265–77. 10.1007/s11154-021-09679-5 PMC8318777 34322844

[48] Jiang Q. Natural forms of vitamin E: metabolism, antioxidant, and anti-inflammatory activities and their role in disease prevention and therapy. Free Radic Biol Med. 2014;72:76–90. 24704972 10.1016/j.freeradbiomed.2014.03.035 PMC4120831

[49] Tosato M, Calvani R, Picca A, Ciciarello F, Galluzzo V, Coelho-Junior HJ, et al. Effects of l-Arginine plus vitamin C supplementation on physical performance, endothelial function, and persistent fatigue in adults with long COVID: A Single-Blind randomized controlled trial. Nutrients. 2022;14:4984. 36501014 10.3390/nu14234984 PMC9738241

[50] Figueiredo LP, Paim P, Cerqueira-Silva T, Barreto CC, Lessa MM. Alpha-lipoic acid does not improve olfactory training results in olfactory loss due to COVID-19: a double-blind randomized trial. Braz J Otorhinolaryngol. 2024;90:101356. 37944311 10.1016/j.bjorl.2023.101356 PMC10665681

[51] Sinopoli A, Sciurti A, Isonne C, Santoro MM, Baccolini Vl. The Efficacy of Multivitamin, Vitamin A, Vitamin B, Vitamin C. and Vitamin D Supplements in the Prevention and Management of COVID-19 and Long-COVID: An Updated Systematic Review and Meta-Analysis of Randomized Clinical Trials. Nutrients. 2024;16:1345. 10.3390/nu16091345 PMC11085542 38732592

[52] Gibson PG, Qin L, Puah SH. COVID-19 acute respiratory distress syndrome (ARDS): clinical features and differences from typical pre-COVID-19 ARDS. Med J Aust. 2020;213:54–6. e51. 32572965 10.5694/mja2.50674 PMC7361309

[53] Kerget B, Cil G, Araz O, Alper F, Akgun M. Comparison of two antifibrotic treatments for lung fibrosis in post-COVID-19 syndrome: A randomized, prospective study. Med Clin (English Edition). 2023;160:525–30. 10.1016/j.medcle.2022.12.019 PMC10273009 37337553

[54] Choudhary R, Kumar A, Ali O, Pervez A. Effectiveness and safety of Pirfenidone and nintedanib for pulmonary fibrosis in COVID-19-Induced severe pneumonia: an interventional study. Cureus. 2022;14:e29435. 36299940 10.7759/cureus.29435 PMC9587348

[55] Wang HY, Tsai SC, Lin YC, Hou JU, Chao CH. The effect of antifibrotic agents on acute respiratory failure in COVID-19 patients: a retrospective cohort study from TriNetX US collaborative networks. BMC Pulm Med. 2024;24:160. 38566026 10.1186/s12890-024-02947-5 PMC10986056

[56] Singh P, Behera D, Gupta S, Deep A, Priyadarshini S, Padhan P. Nintedanib vs Pirfenidone in the management of COVID-19 lung fibrosis: A single-centre study. J Royal Coll Physicians Edinb. 2022;52:100–4. 10.1177/14782715221103402 36146989

[57] Proal AD, VanElzakker MB, Aleman S, Bach K, Boribong BP, Buggert M, et al. SARS-CoV-2 reservoir in post-acute sequelae of COVID-19 (PASC). Nat Immunol. 2023;24:1616–27. 37667052 10.1038/s41590-023-01601-2

[58] Davis HE, McCorkell L, Vogel JM, Topol EJ. Long COVID: major findings, mechanisms and recommendations. Nat Rev Microbiol. 2023;21:133–46. 36639608 10.1038/s41579-022-00846-2 PMC9839201

[59] Jiang J, Li Y, Jiang Q, Jiang Y, Qin H, Li Y. Early use of oral antiviral drugs and the risk of post COVID-19 syndrome: a systematic review and network meta-analysis. J Infect. 2024;106190. 10.1016/j.jinf.2024.106190 38834107

[60] Choi YJ, Seo YB, Seo JW, Lee J, Nham E, Seong H, et al. Effectiveness of antiviral therapy on long COVID: a systematic review and meta-analysis. J Clin Med. 2023;12:7375. 38068427 10.3390/jcm12237375 PMC10707593

[61] Boglione L, Meli G, Poletti F, Rostagno R, Moglia R, Cantone M, et al. Risk factors and incidence of long-COVID syndrome in hospitalized patients: does Remdesivir have a protective effect? QJM. Int J Med. 2021;114:865–71. 10.1093/qjmed/hcab297 PMC8690187 34850210

[62] Fernández-de-las-Peñas C, Franco-Moreno A, Ruiz-Ruigómez M, Arrieta-Ortubay E, Ryan-Murua P, Lumbreras-Bermejo C, et al. Is antiviral treatment with Remdesivir at the acute phase of SARS-CoV-2 infection effective for decreasing the risk of Long-Lasting Post-COVID symptoms?? Viruses. 2024;16:947. 38932239 10.3390/v16060947 PMC11209434

[63] Nevalainen OP, Horstia S, Laakkonen S, Rutanen J, Mustonen JM, Kalliala IE, et al. Effect of Remdesivir post hospitalization for COVID-19 infection from the randomized SOLIDARITY Finland trial. Nat Commun. 2022;13:6152. 36257950 10.1038/s41467-022-33825-5 PMC9579198

[64] Chuang MH, Wu JY, Liu TH, Hsu WH, Tsai YW, Huang PY, et al. Efficacy of nirmatrelvir and Ritonavir for post-acute COVID‐19 sequelae beyond 3 months of SARS‐CoV‐2 infection. J Med Virol. 2023;95:e28750. 37185834 10.1002/jmv.28750

[65] Fung KW, Baye F, Baik SH, McDonald CJ. Nirmatrelvir and molnupiravir and post–COVID-19 condition in older patients. JAMA Intern Med. 2023;183:1404–6. 37870856 10.1001/jamainternmed.2023.5099 PMC10594174

[66] Xie Y, Choi T, Al-Aly Z. Molnupiravir and risk of post-acute sequelae of covid-19: cohort study. BMJ. 2023;381. 10.1136/bmj-2022-074572 PMC10126525 37161995

[67] Liu TH, Chuang MH, Wu JY, Huang PY, Tsai YW, Hsu WH, et al. Effectiveness of oral antiviral agents on long-term cardiovascular risk in nonhospitalized patients with COVID‐19: A multicenter matched cohort study. J Med Virol. 2023;95:e28992. 37522355 10.1002/jmv.28992

[68] Liu TH, Wu JY, Huang PY, Tsai YW, Lai CC. The effect of nirmatrelvir-ritonavir on the long‐term risk of neuropsychiatric sequelae following COVID‐19. J Med Virol. 2023;95:e28951. 37436873 10.1002/jmv.28951

[69] Ioannou GN, Berry K, Rajeevan N, Li Y, Mutalik P, Yan L, et al. Effectiveness of Nirmatrelvir–Ritonavir against the development of Post–COVID-19 conditions among US veterans: A target trial emulation. Ann Intern Med. 2023;176:1486–97. 37903369 10.7326/M23-1394 PMC10620954

[70] Geng LN, Bonilla H, Hedlin H, Jacobson KB, Tian L, Jagannathan P et al. Nirmatrelvir-Ritonavir and symptoms in adults with postacute sequelae of SARS-CoV-2 infection: the STOP-PASC randomized clinical trial. JAMA Intern Med. 2024. 10.1001/jamainternmed.2024.2007 PMC11161857 38848477

[71] Anderson GM, Cook EH, Blakely RD, Sutcliffe JS, Veenstra-VanderWeele J. Long COVID-19 and peripheral serotonin: A commentary and reconsideration. J Inflamm Res. 2024;17:2169–72. 38628604 10.2147/JIR.S456000 PMC11019386

[72] Butzin-Dozier Z, Ji Y, Deshpande S, Hurwitz E, Anzalone AJ, Coyle J, et al. SSRI use during acute COVID-19 and risk of long COVID among patients with depression. BMC Med. 2024;22:445. 39380062 10.1186/s12916-024-03655-x PMC11462648

[73] Sidky H, Hansen KA, Girvin AT, Hotaling N, Michael SG, Gersing K, et al. Assessing the effect of selective serotonin reuptake inhibitors in the prevention of post-acute sequelae of COVID-19. Comput Struct Biotechnol J. 2024;24:115–25. 38318198 10.1016/j.csbj.2023.12.045 PMC10839808

[74] Prasanth MI, Wannigama DL, Reiersen AM, Thitilertdecha P, Prasansuklab A, Tencomnao T, et al. A systematic review and meta-analysis, investigating dose and time of fluvoxamine treatment efficacy for COVID-19 clinical deterioration, death, and Long-COVID complications. Sci Rep. 2024;14:13462. 38862591 10.1038/s41598-024-64260-9 PMC11166997

