# Supplementary material for: Therapeutic options for the treatment of post-acute sequelae of COVID-19: a scoping review
Source: BMC Infect Dis. 2025 May 22;25:731. doi: 10.1186/s12879-025-11131-x (PMC12096624; doi:10.1186/s12879-025-11131-x)
Supplement: Supplementary file 2 — Supplementary Material 2 [file 12879_2025_11131_MOESM2_ESM.docx]

| **Supplementary table 2. Studies of therapeutic options for the treatment of post-acute sequelae of COVID-19** | | | | | | |
| --- | --- | --- | --- | --- | --- | --- |
| **Author** | **Main Symptoms Targeted** | **Country** | **Study Design** | **Regimen** | **Evaluation** | **Outcomes** |
| **Hyperbaric oxygen therapy** | | | | | | |
| Catalogna et al., 2022 [15] | Cognitive symptoms | Israel | Randomized sham-controlled trial  Adult patients  HBOT (n=28) vs. Control (n=28) | **Treatment group:**  40 sessions (2 months),  5 days per week  Each session: 90 min, 100%  O2 at 2 ATA, with 5-min air  breaks every 20 min  Compression and  decompression rates:  1.0 m/min  **Control group:**  40 sessions (2 months),  5 days per week  Each session: 90 min, air at  1.03 ATA (chamber pressure  was raised to 1.2 ATA  during the first five minutes | NeuroTrax  computerized tests  Brief symptom inventory (BSI-18) | Significant improvement in attention and executive function (p<0.001) and changes in hippocampal functional connectivity (p=0.002) and amygdala circuits (p=0.017) |
| Kjellberg et al., 2023 [17] | HBOT Safety | Sweden | Prospective, randomized, double-blind, placebo-controlled, parallel-arms clinical trial  Adult patients  HBOT (n=8) vs. Control (n=12) | **Treatment group:**  Maximum 10 sessions  within 6 weeks  Each session: 90 min, 100%  O2 at 2.4 ATA, with two  5-min air breaks  **Control group:**  Maximum 10 sessions  within 6 weeks  Each session: 90 min, air at  1.35 ATA, with two 5-min  air breaks | Adverse Events | 60% AEs in HBOT group; 20 related to treatment (common: cough, chest pain): no severe adverse events |
| Zilberman et al., 2022 [13] | Cognitive symptoms | Israel | Randomized, double-blind, sham-controlled trial  Adult patients  HBOT (n=37) vs. Control (n=36) | **Treatment group:**  40 sessions (2 months),  5 days per week  Each session: 90 min, 100%  O2 at 2 ATA, with 5-min air  breaks every 20 min  Compression and  decompression rates:  1.0 m/min  **Control group:**  40 sessions (2 months),  5 days per week  Each session: 90 min, air at  1.03 ATA (chamber pressure  was raised to 1.2 ATA  during the first five minutes  of the session along with  circulating air noise  followed by decompression  (0.4 m/min) to 1.03 ATA  during the next  five minutes) | NeuroTrax  computerized tests  MRI-DTI  Self-reported questionnaires | Significant improvements in cognitive domains (p=0.038), energy (p=0.029), sleep (p=0.042), psychiatric (p=0.008), and pain (p=0.001): changes in brain MRI findings |
| Hadanny et al., 2024 [14] | Cognitive, psychiatric, fatigue, sleep and  pain symptoms | Israel | Longitudinal long-term follow-up of a prospective, randomized controlled trial  Adult patients  HBOT (n=37) vs. Control (n=39) | **Treatment group:**  40 daily sessions, five sessions per week within a 2-month period; The HBOT protocol  included breathing 100% oxygen by mask at 2ATA for 90 min with 5-min air breaks every 20 min. Compression/  decompression rates were 1.0 m/min.  **Control group:**  Without any intervention | Short Form Survey (SF‑36)  Brief Symptom Inventory (BSI‑18)  Pittsburgh Sleep Quality Index (PSQI)  Brief Pain Inventory (BPI) | Quality of life improvements sustained at 1-year follow-up: sleep (moderate effect size), psychiatric (large effect size), pain severity (ES=0.69) and interference (ES=0.83) |
| Leitman et al., 2023 [16] | Cardiac function | Israel | Randomized, double-blind, sham-control, clinical trial.  Adult patients  HBOT (n=30) vs. Control (n=30) | **Treatment group:**  40 daily sessions, five sessions per week within a two-month period. 100% oxygen by mask at 2ATA for 90 min with five-minute  air breaks every 20 min. Compression/decompression rates were 1.0 m/minute  **Control group:**  Breathing 21% oxygen by mask at 1.03 ATA for 90 min. | Global Longitudinal Strain (GLS)  Myocardial Work Index (MWI) | Significant improvement in GLS (p<0.001) with a group-by-time interaction for myocardial work indices (p=0.041) |
| **Ivermectin** | | | | | | |
| Hayward et al., 2024 [23] | General symptoms | UK | Randomized, quadruple-blind, parallel-group trial  Adult patients  Ivermectin (n=361) vs. Control (n=378) | **Treatment group:**  390–470 μg/kg per day for 3 days (median 430 μg/kg per day)  **Control group:**  With placebo | Long COVID diagnosed by a medical provider | No significant improvement in recovery, hospital admissions, or longer-term outcomes with ivermectin |
| Bramante et al., 2023 [22] | General symptoms | US | Prospective, randomized, open-label, placebo-controlled, multi-arm trial  Adult patients  Ivermectin (n=2,157) vs. Control (n=3,256) | **Treatment group:**  300–400 μg/kg, taken as one dose daily for 3 days [18 mg daily (6 ×3 mg tablets) for weight 45–64 kg, 24 mg daily (8 ×3 mg tablets) for weight 65–84 kg, and 30 mg daily (10 ×3 mg tablets) for weight ≥84 kg]  **Control group:**  With placebo | Long COVID diagnosed by a medical provider | No effect on cumulative incidence of PASC with ivermectin compared with placebo |
| **Metformin** | | | | | | |
| Bramante et al., 2023 [22] | General symptoms | US | Prospective, randomized, open-label, placebo-controlled, multi-arm trial  Adult patients  Metformin (n=564)  vs. placebo (n=564) | **Treatment group:**  500 mg on day 1, 500 mg twice daily on days 2–5, then 500 mg in the morning and 1000 mg in the evening up to day 14  **Control group:**  With placebo | Long COVID diagnosed by  a medical provider | Significant decrease in the cumulative incidence of long COVID by day 300: 6.3% in metformin group vs 10.4% in placebo group (p=0.012) |
| **Naltrexone** | | | | | | |
| O’Kelly et al., 2022 [34] | General symptoms | Ireland | Single center interventional pre-post cohort study  Adult patients (n=52) | Low-dose naltrexone starting at 1 mg daily in the first month and increasing to 2 mg in the second month | Self-reported questionnaires | Improvements in six of seven parameters, including recovery perception, daily activities, energy, pain, concentration, and sleep (p≤0.001). |
| Isman et al., 2024 [31] | Persistent fatigue | US | Observational open-label pilot study  Adult patients (n=36) | Low-dose naltrexone at 4.5 mg/day and NAD+ supplementation. | Short Form Survey (SF‑36)  Chalder Fatigue Scale | Significant improvements in SF-36 scores and reduced fatigue on the Chalder scale (p<0.001); approximately 52% of participants were classified as responders. |
| Bonilla et al., 2023 [33] | General symptoms | US | Retrospective study  Adult patients (n=59) | Low-dose naltrexone 0.5-6 mg daily, with a median dose of 2 mg); treatment duration ranged from 77 to 255 days (median 143 days). | Self-reported questionnaires including 29 symptoms  Post-COVID-19 Functional Status Scale (PCFS) | Significant improvements in the number and severity of symptoms, with reduced fatigue and better sleep patterns (p<0.050) |
| Hurt et al., 2024 [32] | General symptoms | US | Cross-sectional study  Adult patients (n=536) | Low-dose naltrexone 0.5-6 mg daily, tailored to individual patient needs | Self-reported questionnaires | Reported helpfulness of low-dose naltrexone by 58% of users, with contribution to improved symptom management, particularly in fatigue and brain fog (p<0.001) |
| Tamariz et al., 2024 [29] | General symptoms | US | Retrospective cohort study  Adult patients (n=108)  Naltrexone (n=24) vs. Amitriptyline (n=41) vs. Duloxetine (n=17) vs. Physical therapy (n=26) | **Treatment group:**  Low-dose naltrexone 1.5-4.5 mg daily  **Control group:**  Amitriptyline (10–20 mg daily), duloxetine (20–120 mg daily), and physical therapy | Self-reported questionnaires | 67% improvement rate reported by patients on low-dose naltrexone, with significant effects on fatigue and pain  Significant symptom improvement with low-dose naltrexone compared to physical therapy alone (HR=5.04, p=0.020) |
| **Palmitoylethanolamide** | | | | | | |
| Versace et al., 2023 [36] | Fatigue and cognitive symptoms | Italy | Randomized controlled trial  Adult patients  PEA-LUT (n=17) vs. Control (n=17) | **Treatment group:**  PEA-LUT (700 mg PEA + 70 mg luteolin), administered orally twice a day for eight weeks  **Control group:**  With placebo | LICI (long-interval intracortical inhibition)  SAI (short-latency afferent inhibition)  LTP (long-term potentiation)-like cortical plasticity | Significant increase of LICI (p=0.009) and LTP-like cortical plasticity (p<0.001 and p=0.010 in PEA-LUT group for T1 and T10) |
| Cantone et al., 2024 [39] | Olfactory dysfunction | Italy | Prospective, randomized controlled trial  Adult patients  umPEA-LUT (n=17) vs. Control (n=23) | **Treatment group:**  umPEA-LUT (700 mg PEA + 70 mg luteolin) with daily OT  **Control group:**  Daily OT alone | TDI scores | Significantly improved TDI scores compared to control group (p<0.001) |
| De Luca et al., 2022 [41] | Olfactory dysfunction and mental clouding | Italy | Longitudinal study  Adult patients  PEA-LUT with OT (n=43) vs. PEA-LUT alone (n=16) vs. OT alone (n=10) | **Treatment group:**  PEA-LUT (700 mg PEA + 70 mg luteolin) with or without OT  **Control group:**  Daily OT alone | Sniffin' Sticks test  Parosmia questionnaire  Mini-Mental State Examination (MMSE) | Significant effect on olfactory improvement (p<0.001) in treatment group, with no statistically significant difference between the groups |
| Di Stadio et al.,  2022 [38] | Olfactory dysfunction | Italy | Randomized, double-blinded, placebo-controlled trial  Adult patients  PEA-LUT (n=130) vs. Control (n=55) | **Treatment group:**  umPEA-LUT (700 mg PEA + 70 mg luteolin) with daily OT  **Control group:**  Daily OT with placebo | Sniffin' Sticks test | Significant improvement in olfactory threshold, discrimination, and identification scores (p=0.0001) |
| D’ascanio et al., 2021 [37] | Olfactory dysfunction | Italy | Randomized controlled trial  Adult patients  PEA-LUT (n=7) vs. Control (n=5) | **Treatment group:**  PEA-LUT with daily OT  **Control group:**  Daily OT alone | Sniffin' Sticks assessment | Significant improvement in olfactory threshold, discrimination, and identification scores in (p=0.010) |
| Cenacchi et al., 2024 [40] | Cognitive symptoms | Italy | Retrospective observational study  Adult patients  PEA-LUT (n=26) vs. Control (n=15) | **Treatment group:**  umPEA-LUT (700 mg PEA + 70 mg luteolin) daily for at least 2 months  **Control group:**  Without any intervention | Montreal Cognitive Assessment (MoCA)  Prospective-Retrospective Memory Questionnaire (PRMQ)  Fatigue Severity Scale (FSS) | Significant improvement in MoCA score (p =0.026) and PRMQ (p < 0.001) |
| **Micronutrient supplements** | | | | | | |
| Figueiredo et al., 2024 [49] | Persistent fatigue | Italy | Single-center, randomized, single-blind, placebo-controlled trial  Adult patients  Case (n=23) vs. Control (n=23) | **Treatment group:**  Twice daily oral supplementation with either a combination of 1.66 g L-arginine plus 500 mg liposomal vitamin C  **Control group:**  With placebo | Distance walked on the 6 min walk test after 28 days of intervention  Muscle strength  Endothelial function measuring the brachial artery dilation | Significant increased the 6 min walk distance (p=0.001), greater improvement in handgrip strength (p=0.030), and flow-mediated dilation (p=0.030) |
| Gibson et al., 2020 [50] | Persistent smell loss | Brazil | Randomized, double-blind, placebo-controlled, two-arm trial  Adult patients  Case (n=49) vs. Control (n=51) | **Treatment group:**  Twice daily alpha-lipoic acid 300 mg for 12-weeks,    **Control group:**  With placebo | Butanol test to determine threshold  Olfactory identification test with 8 scents | No significant differences in Connecticut Chemosensory  Clinical Research Center (CCCRC) test (p =0.630), olfactory threshold (p = 0.500), identification score (p = 0.960) and Visual Analog Scale (VAS) score (p = 0.970) |
| **Antifibrotic agents** | | | | | | |
| Kerget et al., 2022 [53] | Chronic respiratory symptoms after COVID-19 pneumonia | Turkey | Prospective, randomized controlled trial  Adult patients  Nintedanib (n=15) vs. Pirfenidone (n=15) | **Nintedanib group**: 300 mg/day  **Pirfenidone group**:  Up to 1800 mg/day titrated over three weeks | Pulmonary function tests (PFTs)  6-minute walk test (6MWT) distances  Oxygen saturation  Radiological scores | Significant improvement in PFT parameters, 6MWT distances, oxygen saturation, and radiological score in both treatments group; nintedanib showed superior improvement in 6MWT and oxygen saturation but had more frequent side effects (e.g., diarrhea in 80% of patients). |
| Choudhary et al., 2022 [54] | Chronic respiratory symptoms after COVID-19 pneumonia | India | Interventional study  Adult patients  Nintedanib (n=2,500) vs. Pirfenidone (n=2,500) | **Nintedanib group:**  150 mg twice daily  **Pirfenidone group:**  Initiated at 600 mg/day and increased to a target dose of 2,400 mg/day over three weeks | Clinical and functional data (PFTs) | Stabilization of lung function over 12 months by both pirfenidone and nintedanib, with no significant differences between groups in PFT parameters (p>0.050) |
| Wang et al., 2024 [55] | Death | Taiwan | Retrospective cohort study  Adult patients  Antifibrotic agents (n=167) vs. Control (n=167) | **Treatment group:**  Nintedanib (150 mg twice daily) or pirfenidone (started at 600 mg/day, titrated up to 2,400 mg/day)    **Control group:**  Without any intervention | Mortality rates | Significant higher 1-year survival rate in antifibrotic group (p<0.001)  Significant reduction in mortality by nintedanib (p<0.013) and not statistically significant improvement by pirfenidone (p = 0.601) |
| Singh et al., 2022 [56] | Radiological findings | India | Prospective observational study  Adult patients  Nintedanib (n=21) vs. Pirfenidone (n=16) vs. Control (n=19) | **Treatment group:**  Nintedanib (300 mg/day in two doses) or pirfenidone (1,800 mg/day in three doses) with steroids    **Control group:**  Prednisolone at 1 mg/kg body weight, tapered over 12 weeks | CT severity scores (CTSS) | Significant improvement in CTSS with nintedanib compared to pirfenidone with steroids and steroids alone (p<0.050)  No significant advantage of pirfenidone over steroids alone |
| **Anti-viral agents** | | | | | | |
| Jiang et al., 2024 [59] | General symptoms | China | Meta-analyses  Included articles (n=9) | **Treatment group:**  NMV-r or Molnupiravir  **Control group:**  Without any intervention | Varies from study to study | Significant reduced PASC risk with early oral antiviral drugs (RR 0.77, p=0.031) |
| Choi et al., 2023 [60] | General symptoms, hospitalization and mortality | South Korea | Meta-analyses  Included articles (n=6) | **Treatment group:**  Remdesivir, NMV-r or Molnupiravir  **Control group:**  Without any intervention | Varies from study to study | Significant reduced occurrence of PASC by 27.5% and PASC-related hospitalization and mortality by 29.7% compared to control group (p<0.050) |
| Boglione et al., 2023 [61] | General symptoms | Italy | Prospective observational study  Adult patients  Remdesivir (n=163) vs. Control (n=165) | **Treatment group:**  Remdesivir  **Control group:**  Without remdesivir | Post-COVID-19 Functional Status Scale (PCFS) | Significant reduction in PASC rate in the remdesivir group (OR=0.64, p<0.001) |
| Fernández et al., 2024 [62] | General symptoms, hospitalization, and ER visit | Italy | Case-control study  Adult patients  Remdesivir (n=216) vs. Control (n=216) | **Treatment group:**  Remdesivir 200 mg the first day and 100 mg/day for 5 consecutive days or until discharge (if shorter than 5 days) for a maximum duration of 10 days  **Control group:**  Without any intervention | Self-reported questionnaires  Anxiety levels (HADS-A)  Depressive symptoms (HADS-D)  Sleep quality (PSQI),  Severity/disability (FIC) | Significant reduction in PASC rate in the remdesivir group (OR=0.40, p<0.001), specifically for fatigue, pain, dyspnea, concentration loss, memory loss, hair loss, and skin rash |
| Nevalainen et al., 2022 [63] | General symptoms | Finland | Parallel 1:1 randomized, open-label trial  Adult patients  Remdesivir (n=98) vs. Control (n=83) | **Treatment group:**  Remdesivir 200 mg on day 1, then 100 mg daily until discharge or maximum 10 days  **Control group:**  Local standard of care | Self-reported questionnaires | No significant difference in quality of life or symptom outcomes between groups, with self-reported recovery rates of 85% and 86%, respectively |
| Chuan et al., 2023 [64] | General symptoms, hospitalization, and ER visit | Taiwan | Retrospective, propensity score-matched, cohort study  Adult patients  NMV-r (n=12,245) vs. Control (n=12,245) | **Treatment group:**  NMV-r treatment within 5 days of COVID-19 diagnosis  **Control group:**  Without any intervention | Diagnostic codes in TriNetX platform | Significant reduced risk of all-cause hospitalization and ER visits (p<0.001)  No significant difference in overall risk of PASC between groups (OR=1.04, p=0.2021) |
| Fung et al., 2023 [65] | General symptoms | US | Observational cohort study  Adult patients  Nirmatrelvir(n=51,658) vs. Molnupiravir (n=8,089) vs. Control (n=253,617) | **Treatment group:**  Nirmatrelvir or molnupiravir  **Control group:**  Without any intervention | Diagnostic codes in Medicare database | 11.8% of patients who received nirmatrelvir developed PASC with HR 0.87 (p<0.001) and 13.7% of patients who received molnupiravir with HR 0.92 (p<0.001) |
| Xie et al., 2023 [66] | General symptoms, hospitalization and mortality | US | Observational cohort study  Adult patients  Molnupiravir (n=11,472) vs. Control (n=217,814) | **Treatment group:**  Molnupiravir use within 5 days of COVID-19 diagnosis  **Control group:**  Without any intervention | Diagnostic codes in Veterans Health Administration data | Significant reduced risk of PASC, post-acute death, and post-acute hospital admission by molnupiravir (p<0.050) |
| Liu et al., 2023 [67] | Major adverse cardiovascular events (MACEs) | Taiwan | Retrospective, propensity score-matched, cohort study  Adult patients  NMV-r (n=80,888) vs. Control (n=80,888) | **Treatment group:**  NMV-r or molnupiravir  **Control group:**  Without any intervention | Diagnostic codes in TriNetX platform | Significant reduced risk of MACE in the antiviral group (HR=0.68, p<0.001) |
| Liu et al., 2023 [68] | Neuropsychiatric sequelae | Taiwan | Retrospective, propensity score-matched, cohort study  Adult patients  NMV-r (n=27,194) vs. Control (n=27,194) | **Treatment group:**  NMV-r  **Control group:**  Without any intervention | Diagnostic codes in TriNetX platform | Significant reduced risk of any neuropsychiatric sequelae in the NMV-r group (OR=0.63, p<0.001) |
| Ioannou et al., 2023 [69] | General symptoms | US | Retrospective, propensity score-matched, cohort, target trial emulation study  Adult patients  NMV-r (n=9.593) vs. Control (n=9.593) | **Treatment group:**  NMV-r treatment within 5 days of COVID-19 diagnosis  **Control group:**  Without any intervention | Diagnostic codes in Veterans Health Administration data | No significant difference in the incidence of most PASC between the two groups, except for lower combined risk for venous thromboembolism and pulmonary embolism |
| Geng et al., 2024 [70] | General symptoms | US | Double-blind, randomized clinical trial  Adult patients with PASC  NMV-r (n=102) vs. Control (n=53) | **Treatment group:**  Nirmatrelvir 300 mg with ritonavir 100 mg, taken orally twice daily for 15 days  **Control group:**  Placebo with ritonavir 100 mg | Self-reported questionnaires | No significant difference in the model-derived severity outcome pooled across the 6 core symptoms at 10 weeks |
| **Selective serotonin reuptake inhibitors** | | | | | | |
| Sidky et al., 2024 [73] | General symptoms | US | Retrospective cohort study  Adult patients  Sigma-1 receptor agonist SSRIs (n=1,521) vs. Non-agonist SSRIs (n=1,803) vs. Control (n=14,584) | **Treatment group:**  Sigma-1 receptor agonist SSRIs (e.g., fluvoxamine, fluoxetine, escitalopram) and non-agonist SSRIs (e.g., sertraline, paroxetine, citalopram  **Control group:**  Without any intervention | Diagnostic codes in national COVID Cohort Collaborative (N3C) | 29% reduction in the risk of PASC by sigma-1 receptor agonist SSRIs (RR=0.70, p<0.001), while 21% reduction by non-agonist SSRIs (RR=0.79, p=0.005) |
| Butzin‑Dozier et al., 2024 [72] | General symptoms | US | Retrospective observational study  Adult patients  SSRI (n=100,803) vs. control (n=201,823) | **Treatment group:**  SSRI (fluoxetine, sertraline, paroxetine, citalopram, and escitalopram), prescribed at least 30 days before COVID-19 diagnosis and continuing through the acute phase of the illness  **Control group:**  Without any intervention | Diagnostic codes in national COVID Cohort Collaborative (N3C) | 7.8% reduction in the risk of PASC by SSRI (RR=0.92, p<0.050) |
| Prasanth et al., 2024 [74] | General symptoms | Thailand, Japan | Meta-analyses  Included articles (n=14) | **Treatment group:**  Fluvoxamine 50-300 mg daily, often divided into two or three doses per day, over 10 to 15 days  **Control group:**  Without any intervention | Recovery percentages of PASC symptoms | Not consistently prevention of PASC development by Fluvoxamine |

**Abbreviations.** HBOT, hyperbaric oxygen therapy; ATA, atmosphere absolute; Palmitoylethanolamide-Luteolin, PEA-LUT; ultramicronized PEA, umPEA-LUT; olfactory training, OT; Montreal Cognitive Assessment, MoCA; Prospective–Retrospective Memory Questionnaire, PRMQ; Fatigue Severity Scale, FSS; NMV-r, ritonavir-boosted nirmatrelvir; MACE, major adverse cardiovascular event
