# Supplementary material for: Therapeutic options for the treatment of post-acute sequelae of COVID-19: a scoping review
Source: BMC Infect Dis. 2025 May 22;25:731. doi: 10.1186/s12879-025-11131-x (PMC12096624; doi:10.1186/s12879-025-11131-x)
Supplement: Supplementary file 3 — Supplementary Material 3 [file 12879_2025_11131_MOESM3_ESM.docx]

| **Treatment type** | **Main targeted symptoms** | **Number of Studies** | **Level of evidence** | **Safety concerns** | **Recommendation** |
| --- | --- | --- | --- | --- | --- |
| Hyperbaric oxygen therapy | Cognitive symptoms | 13 | Low to moderate (observational studies & randomized controlled trials) | Potential adverse events: ear barotrauma, oxygen toxicity | ** |
| Ivermectin | General symptoms | 7 | Moderate (randomized controlled trials) | No major adverse events | * |
| Metformin | General symptoms | 11 | Moderate (randomized controlled trials) | No major adverse events | ** |
| Naltrexone | General symptoms | 6 | Low to moderate (observational studies & randomized controlled trials) | No major adverse events: only mild fatigue and diarrhea | ** |
| Palmitoylethanolamide | Olfactory dysfunction | 8 | Moderate (randomized controlled trials) | No major adverse events | ** |
| Micronutrient supplements | Fatigue and olfactory dysfunction | 64 | High (randomized controlled trials & meta-analysis) | No major adverse events | * |
| Antifibrotic agents | Chronic respiratory symptoms | 5 | Low to moderate (observational studies & randomized controlled trials) | Potential adverse events: diarrhea, nausea/vomiting | *** |
| Anti-viral agents | General symptoms | 67 | High (randomized controlled trials & meta-analysis) | Limited studies | *** |
| Selective serotonin reuptake inhibitors | General symptoms | 19 | High (observational studies & meta-analysis) | Limited studies | ** |

**Supplementary table 3. Summary of therapeutic options for post-acute sequelae of COVID-19**

* Not shown any effect

** Controversial but potential

*** Could be considered as a therapeutic option
